# Association Between Thyroid Hormone Parameters and Renal Biochemical Markers in Patients With Subclinical Hypothyroidism: A Cross-Sectional Study From a Tertiary Care Hospital in Himachal Pradesh

**DOI:** 10.7759/cureus.113132

**Published:** 2026-07-21

**Authors:** Arush Bansal, Jagmeet Singh, Naveen Pahal, Harshdeep Singh, Atul Verma, Sakshi Dahmiwal, Prabh Simranpal

**Affiliations:** 1 General Medicine, All India Institute of Medical Sciences, Bathinda, Bathinda, IND; 2 General Medicine, Dr. Rajendra Prasad Government Medical College, Kangra, IND; 3 Internal Medicine, Maharishi Markandeshwar Medical College and Hospital (MMU), Solan, IND; 4 General Medicine, Civil Hospital, Kurukshetra, IND; 5 Ophthalmology, Deen Dayal Upadhyay Hospital, New Delhi, IND; 6 Emergency Medicine, Civil Hospital, Malout, IND

**Keywords:** hyperuricemia, renal function, serum uric acid, subclinical hypothyroidism, thyroid-stimulating hormone

## Abstract

Introduction

Subclinical hypothyroidism (SCH) is characterized by elevated serum thyroid-stimulating hormone (TSH) levels with normal circulating thyroid hormones and has been associated with metabolic and renal abnormalities. Reduced glomerular filtration rate in hypothyroidism has been proposed to impair uric acid excretion, which may contribute to elevated serum uric acid levels. However, the relationship between serum uric acid levels and thyroid function in SCH remains inconclusive. This study aimed to evaluate serum uric acid levels and their correlation with thyroid hormone parameters in patients with SCH.

Materials and methods

This hospital-based cross-sectional observational study was conducted in the Department of General Medicine, Maharishi Markandeshwar Medical College and Hospital, Kumarhatti, Himachal Pradesh, from January 2023 to June 2024. Eighty adults with confirmed SCH (TSH 6.0-9.9 µIU/mL with normal T3 and T4) were enrolled. Serum uric acid was measured using the Roche COBAS c311 clinical chemistry analyzer, while T3, T4, and TSH were estimated using the Roche COBAS e411 immunoassay analyzer. Statistical analysis was performed using SPSS version 26. Independent samples t-test, Chi-square test, and Pearson's correlation analysis were applied, with p<0.05 considered statistically significant.

Results

The mean age of participants was 47.98±14.07 years, and 53 (66.3%) were women. The mean serum T3, T4, TSH, creatinine, and uric acid levels were 1.28±0.32 ng/mL, 6.95±1.23 µg/dL, 6.68±0.56 µIU/mL, 0.75±0.20 mg/dL, and 5.71±1.49 mg/dL, respectively. Hyperuricemia was observed in 24 (30.0%) patients. Weak negative correlations were found between T3 and serum uric acid (r=-0.019) and creatinine (r=-0.063). T4 demonstrated a weak positive correlation with serum uric acid (r=0.110) and a weak negative correlation with creatinine (r=-0.030). TSH showed weak positive correlations with serum uric acid (r=0.017) and creatinine (r=0.078). None of these correlations reached statistical significance (all p>0.05).

Conclusion

Elevated serum uric acid levels were observed in a subset of patients with subclinical hypothyroidism, whereas most participants had normal serum creatinine levels. Although weak correlations were observed between thyroid hormone parameters and renal biochemical markers, none reached statistical significance. Therefore, this study did not detect evidence of a statistically significant linear association between thyroid hormone parameters and renal biochemical markers. Further large-scale multicenter prospective studies incorporating euthyroid controls and adjustment for potential confounding factors are warranted to clarify the clinical significance of these findings.

## Introduction

Subclinical hypothyroidism (SCH) is a common endocrine disorder characterized by elevated serum thyroid-stimulating hormone (TSH) levels with normal circulating thyroid hormone concentrations (free triiodothyronine (T3) and thyroxine (T4)) [1]. The prevalence of SCH has been reported to range from 3% to 15% in the general population, with a higher frequency among women and older adults [2]. Although patients with SCH are often asymptomatic, increasing evidence suggests that even mild thyroid dysfunction may be associated with adverse metabolic and cardiovascular outcomes, including dyslipidemia, endothelial dysfunction, hypertension, coronary artery disease, heart failure, and progression to overt hypothyroidism [3,4]. Therefore, early identification of metabolic alterations observed in patients with SCH is clinically important.

Thyroid hormones play an essential role in regulating basal metabolic rate, protein and lipid metabolism, renal hemodynamics, and glomerular filtration rate (GFR) [5]. Hypothyroidism has been associated with reduced renal plasma flow and GFR, which may impair the renal clearance of several metabolites, including uric acid [6]. Hyperuricemia, defined according to commonly used laboratory reference ranges as serum uric acid levels >7.0 mg/dL in men and >6.0 mg/dL in women, may therefore be observed in some patients with thyroid dysfunction, although multiple factors influence serum uric acid concentrations [7,8]. Elevated serum uric acid has also been recognized as an independent risk factor for gout, chronic kidney disease, metabolic syndrome, hypertension, and cardiovascular disease, making it a clinically relevant biochemical marker for evaluation in patients with endocrine disorders [9].

Several studies have reported an association between thyroid dysfunction and altered serum uric acid levels; however, the relationship remains inconsistent, particularly in patients with subclinical hypothyroidism [10,11]. The proposed mechanisms include reduced renal uric acid clearance secondary to decreased GFR, alterations in insulin resistance, and metabolic changes associated with thyroid hormone deficiency [12,13]. Nevertheless, these mechanisms have not been conclusively established in patients with SCH. While overt hypothyroidism has been consistently associated with hyperuricemia, evidence regarding serum uric acid levels in SCH remains limited and inconsistent, especially in the Indian population. Furthermore, the correlation between serum uric acid and thyroid hormone parameters (T3, T4, and TSH) has not been conclusively established.

Therefore, the present study aimed to evaluate renal biochemical parameters, including serum uric acid and serum creatinine, in patients with subclinical hypothyroidism and to assess their correlation with thyroid hormone parameters (T3, T4, and TSH) among patients attending a tertiary care hospital in Himachal Pradesh.

## Materials and methods

Study design and participants

This hospital-based cross-sectional observational study was conducted in the Department of General Medicine at Maharishi Markandeshwar Medical College and Hospital, Kumarhatti, Solan, Himachal Pradesh, over a period of 1.5 years from January 2023 to June 2024. The study protocol was approved by the Institutional Ethics Committee of Maharishi Markandeshwar Medical College and Hospital (IEC No. MMMCH/IEC/22/599). Written informed consent was obtained from all participants prior to enrolment, and confidentiality of participant information was maintained throughout the study. Adult patients attending the outpatient and inpatient departments who fulfilled the eligibility criteria were recruited by consecutive sampling during the study period. Patients diagnosed with subclinical hypothyroidism were screened for eligibility before enrolment.

Sample size calculation

The sample size was estimated based on the correlation coefficient reported by Shrivatsava et al. (r=0.3915) between serum thyroxine (T4) and serum uric acid levels among patients with hypothyroidism [11]. Although the primary objective of the present study was to evaluate the correlation between thyroid hormone parameters and renal biochemical markers, this published correlation coefficient was used because comparable estimates for patients with subclinical hypothyroidism were not available in the literature.

The sample size for Pearson's correlation analysis was calculated using the formula:

\[ n=\left(\frac{Z_{\alpha/2}+Z_{\beta}} {\tfrac{1}{2}\ln\left(\frac{1+r}{1-r}\right)}\right)^2+3 \]

where $Z_{\alpha/2} = 1.96$ for a 95% confidence level, $Z_{\beta}=0.84$ for 80% power, and r=0.3915.

The calculated minimum sample size was 49 participants. To improve the precision of the estimates and compensate for possible incomplete data or participant exclusions, the target sample size was increased to 80 participants before study commencement.

Eligibility criteria

Patients aged 18 years or older with confirmed SCH, defined as serum TSH levels between 6.0 and 9.9 µIU/mL with normal serum T3 and T4 concentrations, were enrolled irrespective of sex.

The TSH range of 6.0-9.9 µIU/mL was selected to include patients with biochemically established mild subclinical hypothyroidism while excluding overt hypothyroidism requiring treatment.

The detailed inclusion and exclusion criteria are presented in Table 1 [14].

**Table 1:** Inclusion and Exclusion Criteria TSH: thyroid-stimulating hormone; T3: triiodothyronine; T4: thyroxine.

Inclusion Criteria	Exclusion Criteria
Age ≥18 years	Age <18 years
Confirmed subclinical hypothyroidism	Pregnancy
Serum TSH 6.0-9.9 µIU/mL	Lactation
Normal serum T3 concentration	Overt hypothyroidism (TSH ≥10 µIU/mL or abnormal T3/T4 levels)
Normal serum T4 concentration	Previous or current thyroxine therapy
Either sex	Thyrotoxicosis
	Chronic kidney disease
	Autosomal dominant polycystic kidney disease
	Malignancy
	Receiving chemotherapy
	Receiving radiotherapy
	Receiving uric acid-lowering medications

Laboratory investigations

Venous blood samples were collected under standard aseptic precautions for biochemical analysis. Serum uric acid levels were measured using the Roche COBAS c311 clinical chemistry analyzer (Roche Diagnostics, Mannheim, Germany). Thyroid function tests, including total serum triiodothyronine (T3), total thyroxine (T4), and thyroid-stimulating hormone (TSH), were analyzed using the Roche COBAS e411 immunoassay analyzer (Roche Diagnostics, Mannheim, Germany). All biochemical analyses were performed according to the manufacturer's instructions under routine internal quality-control procedures followed by the institutional central laboratory. The laboratory reference ranges were 0.84-2.02 ng/mL for T3, 5.13-14.06 µg/dL for T4, and 0.27-4.20 µIU/mL for TSH. Hyperuricemia was defined as a serum uric acid concentration >7.0 mg/dL in males and >6.0 mg/dL in females, according to the institutional laboratory reference ranges [7].

Statistical analysis

All collected data were entered into a structured database and analyzed using the Statistical Package for the Social Sciences (SPSS) version 26 (IBM Corp., Armonk, NY, USA). Data were reviewed for completeness before statistical analysis, and participants with incomplete biochemical measurements were not included in the final analysis. Continuous variables were expressed as mean±standard deviation (SD), whereas categorical variables were presented as frequency and percentage (n(%)). The normality of continuous variables was assessed before applying parametric tests. Comparisons between two groups were performed using the independent samples t-test for continuous variables. Categorical variables were compared using the chi-square test or Fisher's exact test, where appropriate. Pearson's correlation coefficient was used to evaluate the association between thyroid hormone parameters and serum uric acid and creatinine levels. All statistical tests were two-tailed, and a p-value <0.05 was considered statistically significant.

## Results

A total of 80 patients with subclinical hypothyroidism were included in the study. The mean age of the study population was 47.98±14.07 years. Of the total participants, 27 (33.8%) were men and 53 (66.3%) were women, indicating a female predominance among patients with subclinical hypothyroidism (Table 2).

**Table 2:** Baseline demographic characteristics of the study population (N=80) Data are presented as mean±standard deviation (SD) for continuous variables and number (percentage) (n (%)) for categorical variables. Descriptive statistics were used for data summarization. Abbreviations: N, total study population; n, number of participants; SD, standard deviation.

Variable	Value
Age (years), mean±SD	47.98±14.07
Male, n (%)	27 (33.8%)
Female, n (%)	53 (66.3%)

The mean age of male participants was 52.07±12.93 years, compared with 45.90±14.28 years among female participants. Although men had a higher mean age than women, this difference was not statistically significant (t=1.90, p=0.06) (Table 3).

**Table 3:** Comparison of age between male and female participants Data are presented as mean±standard deviation (SD). Comparison between males and females was performed using the independent Student's t-test. A p-value <0.05 was considered statistically significant. Abbreviations: SD, standard deviation; t, Student's t-test statistic.

Variable	Males (n=27)	Females (n=53)	Mean difference (95% CI)	t	p-value
Age (years)	52.07±12.93	45.90±14.28	6.17 (-0.29 to 12.63)	1.90	0.06

The mean serum T3, T4, and TSH levels among the study participants were 1.28±0.32 ng/mL, 6.95±1.23 µg/dL, and 6.68±0.56 µIU/mL, respectively. Serum T3 levels ranged from 0.84 to 2.01 ng/mL, T4 levels ranged from 5.23 to 11.36 µg/dL, and TSH levels ranged from 6.00 to 8.55 µIU/mL (Table 4).

**Table 4:** Thyroid hormone profile of the study population Data are presented as mean±standard deviation (SD) and minimum-maximum (range). Descriptive statistics were used for data summarization. Abbreviations: SD, standard deviation; T3, triiodothyronine; T4, thyroxine; TSH, thyroid-stimulating hormone.

Parameter	Mean±SD	Minimum	Maximum
T3 (ng/mL)	1.28±0.32	0.84	2.01
T4 (µg/dL)	6.95±1.23	5.23	11.36
TSH (µIU/mL)	6.68±0.56	6.00	8.55

Male participants had a mean serum T3 level of 1.27±0.31 ng/mL, whereas female participants had a mean serum T3 level of 1.23±0.33 ng/mL; the difference was not statistically significant (t=0.51, p=0.61). Similarly, the mean serum T4 level was 6.73±0.99 µg/dL in males and 7.07±1.34 µg/dL in females (t=-1.18, p=0.24). The mean serum TSH level was 6.68±0.60 µIU/mL in men and 6.68±0.55 µIU/mL in women, with no statistically significant difference between the two groups (t=0.05, p=0.96) (Table 5).

**Table 5:** Comparison of thyroid hormone profile between male and female participants Data are presented as mean±standard deviation (SD). Comparison between males and females was performed using the Independent Student's t-test. A p-value <0.05 was considered statistically significant. Abbreviations: SD, standard deviation; T3, triiodothyronine; T4, thyroxine; TSH, thyroid-stimulating hormone; t, Student's t-test statistic.

Parameter	Males (n=27)	Females (n=53)	Mean difference (95% CI)	t	p-value
T3 (ng/mL)	1.27±0.31	1.23±0.33	0.04 (-0.12 to 0.20)	0.51	0.61
T4 (µg/dL)	6.73±0.99	7.07±1.34	-0.34 (-0.91 to 0.23)	-1.18	0.24
TSH (µIU/mL)	6.68±0.60	6.68±0.55	0.00 (-0.26 to 0.26)	0.05	0.96

Among the study population, 76 (95.0%) participants had normal serum creatinine levels, while 4 (5.0%) had elevated serum creatinine levels. The overall mean serum creatinine concentration was 0.75±0.20 mg/dL, with values ranging from 0.24 to 1.30 mg/dL. Regarding serum uric acid, 56 (70.0%) participants had normal levels, whereas 24 (30.0%) had hyperuricemia. The mean serum uric acid level was 5.71±1.49 mg/dL, with a minimum value of 1.40 mg/dL and a maximum value of 8.60 mg/dL (Table 6).

**Table 6:** Distribution of renal biochemical parameters among the study population (N = 80) Data are presented as number (percentage) [n (%)] for categorical variables and mean ± standard deviation (SD) with minimum–maximum (range) for continuous variables. Descriptive statistics were used for data summarization. *Hyperuricemia was defined as serum uric acid >7.0 mg/dL in males and >6.0 mg/dL in females, based on the institutional laboratory reference ranges. Abbreviations: N, total study population; n, number of participants; SD, standard deviation.

Parameter	Category	Value
Serum creatinine (mg/dL)	Normal (<1.2)	76 (95.0%)
	Elevated (≥1.2)	4 (5.0%)
	Mean±SD	0.75 ± 0.20
	Minimum	0.24
	Maximum	1.30
Serum uric acid (mg/dL)	Normal	56 (70.0%)
	Hyperuricemia*	24 (30.0%)
	Mean±SD	5.71 ± 1.49
	Minimum	1.40
	Maximum	8.60

Normal serum creatinine levels were observed in 25 (92.6%) male participants and 51 (96.2%) female participants, whereas elevated serum creatinine levels were present in two (7.4%) men and two (3.8%) women. The distribution of serum creatinine categories did not differ significantly between the sexes (p=0.49). Similarly, normal serum uric acid levels were observed in 20 (74.1%) men and 36 (67.9%) women, while hyperuricemia was present in seven (25.9%) men and 17 (32.1%) women. The study did not detect a statistically significant association between sex and serum uric acid status (χ²=0.34, p=0.56) (Table 7).

**Table 7:** Sex-wise comparison of renal biochemical parameters Data are presented as number (percentage) (n (%)). Comparisons between men and women were performed using the chi-square (χ²) test or Fisher's exact test. A p-value <0.05 was considered statistically significant. Abbreviations: n, number of participants; χ², Chi-square statistic; df, degree of freedom.

Parameter	Category	Males (n=27)	Females (n=53)	χ² (df)	p-value
Serum creatinine	Normal	25 (92.6%)	51 (96.2%)	Fisher's exact test	0.49
	Elevated	2 (7.4%)	2 (3.8%)		
Serum uric acid	Normal	20 (74.1%)	36 (67.9%)	0.34 (1)	0.56
	Hyperuricemia	7 (25.9%)	17 (32.1%)		

Pearson correlation analysis demonstrated weak correlations between thyroid hormone parameters and renal biochemical markers. Serum T3 showed a weak negative correlation with serum uric acid (r=-0.019, p=0.865) and serum creatinine (r=-0.063, p=0.580). Serum T4 exhibited a weak negative correlation with serum creatinine (r=-0.030, p=0.790) and a weak positive correlation with serum uric acid (r=0.110, p=0.331). Likewise, serum TSH demonstrated weak positive correlations with serum uric acid (r=0.017, p=0.880) and serum creatinine (r=0.078, p=0.491). None of these correlations reached statistical significance, and the study did not detect evidence of a linear association between thyroid hormone levels and renal biochemical parameters in this study population (Table 8).

**Table 8:** Pearson correlation between thyroid hormone levels and renal biochemical parameters Data are presented as Pearson's correlation coefficient (r) and p-value. Correlations were assessed using Pearson's correlation analysis. A p-value <0.05 was considered statistically significant. Abbreviations: T3, triiodothyronine; T4, thyroxine; TSH, thyroid-stimulating hormone; r, Pearson's correlation coefficient.

Variables	Pearson's r	95% CI	p-value
T3 vs Serum uric acid	-0.019	-0.237 to 0.202	0.865
T3 vs Serum creatinine	-0.063	-0.277 to 0.160	0.580
T4 vs Serum creatinine	-0.030	-0.248 to 0.191	0.790
T4 vs Serum uric acid	0.110	-0.112 to 0.321	0.331
TSH vs Serum uric acid	0.017	-0.202 to 0.236	0.880
TSH vs Serum creatinine	0.078	-0.145 to 0.292	0.491

## Discussion

The present study evaluated serum uric acid levels and their relationship with thyroid function in patients with subclinical hypothyroidism. Thyroid hormones play a crucial role in maintaining renal hemodynamics. Although overt hypothyroidism is well recognized to affect renal function, the impact of subclinical hypothyroidism remains less clearly defined. In the present study, the mean age of the study population was 47.98±14.07 years, with the majority of participants belonging to the middle-aged group, which is comparable to findings reported in previous studies. Deshmukh et al. observed that nearly 148 (62.5%) of patients with subclinical hypothyroidism belonged to the 35- to 54-year age group, while Tunbridge et al. reported a mean age of 47.10±16.40 years among patients with subclinical hypothyroidism [15,16]. Furthermore, women constituted 53 (66.3%) of the study population, confirming the higher prevalence of subclinical hypothyroidism among women. Similar findings were reported by Bashir et al., who demonstrated a significantly greater prevalence of subclinical hypothyroidism in women than in men [17]. The demographic profile observed in the present study is therefore consistent with previously published hospital-based studies involving patients with subclinical hypothyroidism.

The thyroid profile observed in the present study was consistent with the biochemical characteristics of subclinical hypothyroidism. The mean serum T3, T4, and TSH levels were 1.28±0.32 ng/mL, 6.95±1.23 µg/dL, and 6.68±0.56 µIU/mL, respectively, with no significant sex-based differences. Gupta et al. reported a mean serum T3 level of 0.96±0.17 ng/mL, while Singh et al. observed a mean TSH concentration of 8.93±0.96 µIU/mL in hypothyroid patients, findings that are broadly comparable with the biochemical profile observed in the present study [18,19]. However, these comparisons should be interpreted cautiously because differences in study populations, diagnostic criteria, and disease severity may influence thyroid hormone profiles.

Regarding renal biochemical parameters, the mean serum uric acid level was 5.71±1.49 mg/dL, and 24 (30.0%) participants had hyperuricemia. Although serum creatinine remained within the normal range in most patients, four (5.0%) participants had elevated creatinine levels. These findings indicate that renal biochemical abnormalities were present in a proportion of patients with subclinical hypothyroidism; however, in the absence of a euthyroid control group, it cannot be determined whether these abnormalities were attributable to thyroid dysfunction.

Correlation analysis demonstrated weak associations between thyroid hormone parameters and renal biochemical markers. Serum T3 showed weak negative correlations with serum uric acid (r=-0.019) and serum creatinine (r=-0.063), whereas serum T4 demonstrated a weak positive correlation with serum uric acid (r=0.110) and a weak negative correlation with serum creatinine (r=-0.030). Likewise, serum TSH exhibited weak positive correlations with serum uric acid (r=0.017) and serum creatinine (r=0.078); however, none of these correlations reached statistical significance. Similar inverse relationships between serum T3 and creatinine have been reported by Naguib et al., whereas Tayal et al. also demonstrated a negative correlation between serum T3 and serum uric acid, although the strength of association was greater in their study (r=-0.29) [20,21]. Likewise, Singh et al. reported a negative association between serum T4 and creatinine, while Boruah et al. demonstrated a positive correlation between serum T4 and serum uric acid, which was similar in direction to the findings of the present study but differed in magnitude and statistical significance [8,19].

Previous investigators have suggested that increasing TSH levels may be associated with rising serum uric acid and creatinine concentrations because reduced thyroid hormone activity has been proposed to reduce renal blood flow, lower GFR, and impair renal urate clearance. Saini et al. and Tayal et al. reported significant positive correlations between serum TSH and both serum uric acid and creatinine, whereas the present study demonstrated only weak positive correlations that did not reach statistical significance [21,22]. The discrepancy may reflect differences in sample size, patient characteristics, severity and duration of thyroid dysfunction, study design, and inclusion criteria across studies. Given the very small correlation coefficients observed in the present study (r ranging from -0.063 to 0.110), the findings do not provide evidence of a statistically significant linear association between thyroid hormone parameters and renal biochemical markers. Although the observed directions of correlation are comparable to those reported in some previous studies, these findings should be interpreted cautiously and should not be considered evidence of a biological relationship. Further large-scale, multicenter prospective studies incorporating euthyroid controls, assessment of potential confounding factors, and longitudinal follow-up are warranted to clarify the relationship between thyroid function and renal biochemical parameters in patients with subclinical hypothyroidism.

Strengths and limitations

The present study has several strengths. It specifically evaluated serum uric acid levels in patients with subclinical hypothyroidism, a relatively underexplored area, particularly in the Indian population. Standardized biochemical analyses were performed using automated Roche COBAS analyzers, ensuring reliable laboratory measurements. Additionally, strict inclusion and exclusion criteria minimized the influence of important clinical conditions such as chronic kidney disease, overt hypothyroidism, pregnancy, malignancy, and the use of uric acid-lowering medications, thereby improving the internal validity of the study.

However, certain limitations should be acknowledged. This was a single-center hospital-based cross-sectional study with a relatively modest sample size, which may limit the generalizability of the findings. The absence of a euthyroid control group precluded direct comparison of serum uric acid levels between individuals with and without subclinical hypothyroidism. Furthermore, the cross-sectional design does not permit the establishment of causal relationships or assessment of temporal changes in serum uric acid following treatment of subclinical hypothyroidism. In addition, eGFR was not assessed, and thyroid function was evaluated using a single measurement without repeat testing. Potential confounding factors affecting serum uric acid levels, such as dietary purine intake, alcohol consumption, body mass index, insulin resistance, comorbid conditions, concomitant medications, menopausal status, hydration status, and serum thyroid autoantibody status, were not evaluated. Therefore, larger multicentric prospective studies with adequate adjustment for these potential confounders are warranted to validate these findings and further elucidate the relationship between thyroid dysfunction and renal biochemical parameters.

## Conclusions

The present study demonstrated that serum uric acid levels were elevated in a subset of patients with subclinical hypothyroidism, whereas serum creatinine levels remained within the normal range in most participants. Weak positive correlations were observed between serum TSH and serum uric acid and creatinine, while serum T3 and T4 showed weak inverse or positive associations with renal biochemical parameters; however, none of these correlations reached statistical significance. Therefore, the present study did not detect evidence of a statistically significant linear association between thyroid hormone parameters and renal biochemical markers. Although elevated serum uric acid levels were observed in some patients, the absence of a euthyroid control group precludes conclusions regarding their relationship with subclinical hypothyroidism. Further large-scale multicenter prospective studies incorporating euthyroid controls, adjustment for potential confounding factors, and longitudinal follow-up are warranted to clarify the relationship between thyroid function and renal biochemical parameters and to determine the clinical significance of these findings.
